# Small Neotropical primates promote the natural regeneration of anthropogenically disturbed areas

**DOI:** 10.1038/s41598-019-46683-x

**Published:** 2019-07-25

**Authors:** Eckhard W. Heymann, Laurence Culot, Christoph Knogge, Andrew C. Smith, Emérita R. Tirado Herrera, Britta Müller, Mojca Stojan-Dolar, Yvan Lledo Ferrer, Petra Kubisch, Denis Kupsch, Darja Slana, Mareike Lena Koopmann, Birgit Ziegenhagen, Ronald Bialozyt, Christina Mengel, Julien Hambuckers, Katrin Heer

**Affiliations:** 1 Verhaltensökologie & Soziobiologie, Deutsches Primatenzentrum – Leibniz-Institut für Primatenforschung, Göttingen, Germany; 2 Laboratório de Primatologia, Departamento de Zoologia, Universidade Estadual Paulista - UNESP, Rio Claro, SP Brazil; 3 Primatology Research Group, Behavioral Biology Unit, University of Liège, Liège, Belgium; 4 School of Life Sciences, Anglia Ruskin University, Cambridge, UK; 5 Facultad de Ciencias Biológicas, Universidad Nacional de la Amazonía Peruana, Iquitos, Peru; 6 Present Address: Bayerisches Landesamt für Gesundheit und Lebensmittelsicherheit, Erlangen, Germany; 7 Facultad de Psicología, Universidad Autónoma, Madrid, Spain; 8 Present Address: Albrecht-von-Haller-Institut für Pflanzenwissenschaften, Abteilung Ökologie & Ökosystemforschung, Georg-August Universität Göttingen, Göttingen, Germany; 9 Present Address: Naturschutzbiologie, Georg-August Universität Göttingen, Göttingen, Germany; 10 Present Address: Bioplan Marburg, Marburg, Germany; 11 Naturschutzbiologie, Philipps-Universität Marburg, Marburg, Germany; 12 Present Address: Nordwestdeutsche Forstliche Versuchsanstalt, Göttingen, Germany; 13 Chair for Statistics and Econometrics, Georg-August Universität Göttingen, Göttingen, Germany; 14 Present Address: Department of Finance, HEC Liège, University of Liège, Liège, Belgium

**Keywords:** Tropical ecology, Tropical ecology, Restoration ecology

## Abstract

Increasingly large proportions of tropical forests are anthropogenically disturbed. Where natural regeneration is possible at all, it requires the input of plant seeds through seed dispersal from the forest matrix. Zoochorous seed dispersal – the major seed dispersal mode for woody plants in tropical forests – is particularly important for natural regeneration. In this study, covering a period of more than 20 years, we show that small New World primates, the tamarins *Saguinus mystax* and *Leontocebus nigrifrons*, increase their use of an anthropogenically disturbed area over time and disperse seeds from primary forest tree species into this area. Through monitoring the fate of seeds and through parentage analyses of seedlings of the legume *Parkia panurensis* from the disturbed area and candidate parents from the primary forest matrix, we show that tamarin seed dispersal is effective and contributes to the natural regeneration of the disturbed area.

## Introduction

The extent of tropical forests is declining worldwide^1^ and in the Neotropics, moist forests suffer the strongest net loss^2^. Where forests are not irreversibly destroyed (through construction of hydroelectric dams, roads etc.) or converted into permanent agricultural areas, deforested areas may regenerate into old-growth forest through various phases of secondary growth^3,4^. This regeneration process is promoted by seed dispersal into deforested areas^5,6^ with the importance of zoochorous dispersal by frugivorous animals increasing with the age of the regenerating area^7,8^. In early stages of regeneration, flying vertebrates – bats and birds – may drop seeds in defecations while flying over the area or roosting in remnant trees^9,10^. With increasing tree cover, arboreal mammals like primates can visit and disperse seeds into regenerating areas.

Tamarins, small Neotropical primates from the family Callitrichidae, are tolerant to high levels of forest disturbance. They use, persist in or might even prefer secondary forest^11–14^. Tamarins are highly frugivorous, disperse seeds from a broad spectrum of plant species^15–17^, and also disperse seeds into secondary forests^18,19^. While there is evidence for seasonality in the use of secondary forests^19,20^, long-term trends or patterns in tamarins’ use of secondary forest, particularly in early stages of regeneration have not been examined. This is, however, relevant for evaluating the contribution they can make to natural regeneration of such areas. Therefore, in this study we examine (1) how the use of an anthropogenically degraded (clear-cut for buffalo pasture), but regenerating area (in the following we use the term “secondary forest” for this area) by tamarins changes over time; (2) which plant species attract tamarins to the secondary forest; and (3) whether seed dispersal into the secondary forest contributes to natural regeneration.

We use long-term ranging and feeding data from a population of two sympatric tamarin species, *Leontocebus nigrifrons* (previously *Saguinus fuscicollis nigrifrons*; see^21,22^ and *Saguinus mystax*, for examining the first and second question. The third question is tackled by (a) following the fate of tamarin-dispersed seeds from a diversity of plant species in the secondary forest; and (b) genotyping seedlings of a tamarin-dispersed tree species, *Parkia panurensis*, growing in the secondary forest and from adults growing the adjacent primary forest and subsequent parentage analyses. This plant species is particularly suitable for examining the role of tamarins for regeneration, as these primates are the exclusive seed dispersers of *P. panurensis* at our study site.

## Results

### Temporal patterns in the use of the secondary forest

**Figure 1:**
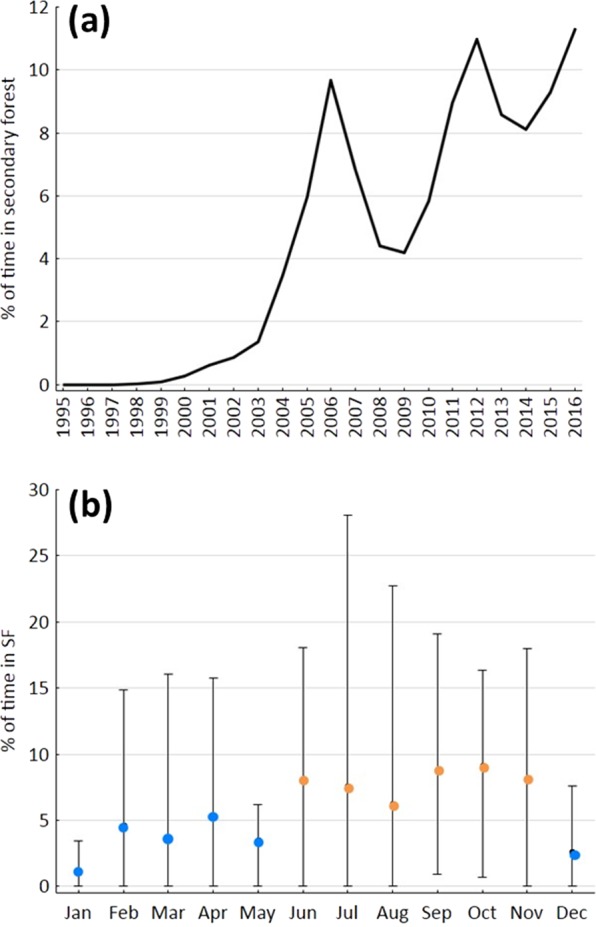
Temporal patterns of secondary forest use by the tamarins. (**a**) Long-term pattern (yearly means), corrected for uneven representation of months and potential seasonal variation. (**b**) Yearly pattern (●: monthly means; whiskers: monthly minima and maxima). Blue dots: months with ≥250 mm of rainfall; orange dots: months with <250 mm of rainfall.

**Figure 2:**
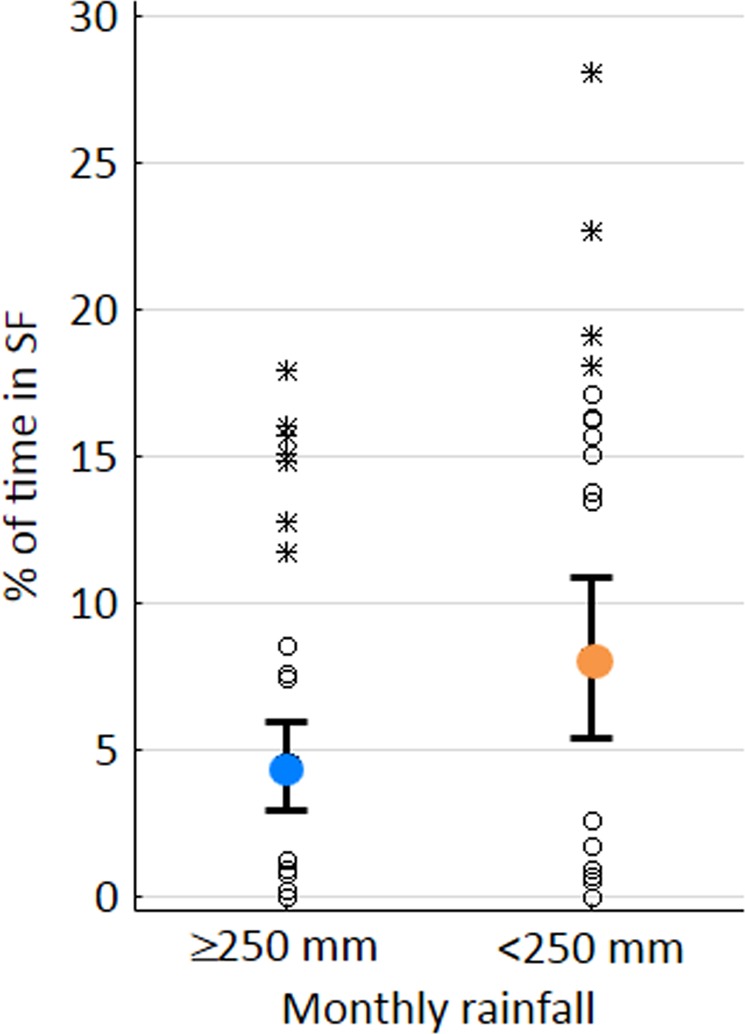
Use of secondary forest in months with ≥250 mm of rainfall and <250 mm of rainfall. ●: means; ○: outliers; *extremes.

Tamarins were first observed to enter the secondary forest in 2000, i.e. the year in which the pasture was abandoned. Between 2000 and 2003, they spent generally <1.5% of the yearly activity period in secondary forest; thereafter, time in the secondary forest strongly increased but showed fluctuations with pronounced peaks in 2006 and 2012 and lows in 2008 and 2009 (Fig. 1a). Although time in the secondary forest tended to be higher from June to November (Fig. 1b), monthly variation was not significant (ANOVA: F_1,11_ = 1.022, n.s.). The proportion of time in the secondary forest was on average two times higher in months with <250 mm compared to months with ≥250 mm rainfall (Fig. 2), but the difference was only marginally significant (t_78_ = −1.951, p = 0.055).

### Feeding patterns in the secondary forest

A total of 31 plant species were exploited for fruit in the secondary forest (Supplementary Information [Supplementary Table 1]). This corresponded to ca. 10% of the total number of plant species exploited by tamarins for fruit, nectar or exudate. While in 2000 only one fruit species was consumed in the secondary forest (*Miconia ternatifolia*), the six fruit species were consumed in 2005 (4 observation months), 12 in 2006 (8 observation months), 16 in 2007 (7 observation months), and 19 in 2008 (6 observation months).

### Seedling recruitment in the secondary forest

We followed the fate of 487 identified seeds for at least one year; 47 seeds (9.6%) were dispersed from primary into secondary forest. Of these 47, 15 (31.9%) seeds dispersed into secondary forest survived for at least one year and germinated. In comparison, 82 out of 440 (18.6%) seeds dispersed in primary forest recruited and survived for at least one year. Recruited seedlings in secondary forest belonged to eight species: *Inga acrocephala, Inga lopadadenia, Inga umbellifera, Parkia panurensis* (all Fabaceae); *Dicranostyles* aff. *scandens* (Convolvulaceae), *Naucleopsis mello-barretoi* (Moraceae); *Paullinia sp*. (Sapindaceae)*, Micropholis egensis* (Sapotaceae). Adults of all species except *I. lopadadenia* were only present in primary forest.

### Recruitment of *Parkia panurensis* in the secondary forest

**Figure 3:**
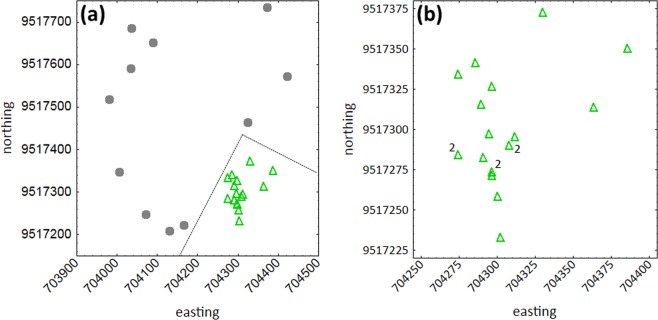
*Parkia panurensis* seedling. ©Eckhard W. Heymann, 2016.

Seedlings (Fig. 3) and juveniles of *P. panurensis* in the secondary forest ranged from 15 to 210 cm in height, suggesting they originated from seeds dispersed in different years.

**Figure 4:**
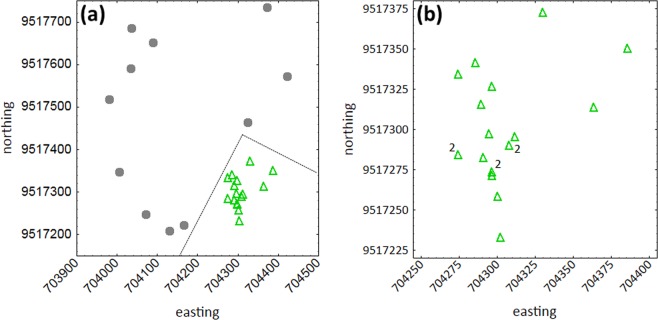
(**a**) Locations of seedlings and juveniles (green triangles) in the secondary forest and of candidate parents (grey dots) in the primary forest. Coordinates are UTM–WGS 84 for cell 18. (**b**) Enlarged view of the position of seedlings and juveniles. The number indicates the location of two seedlings at the same GPS position.

**Figure 5:**
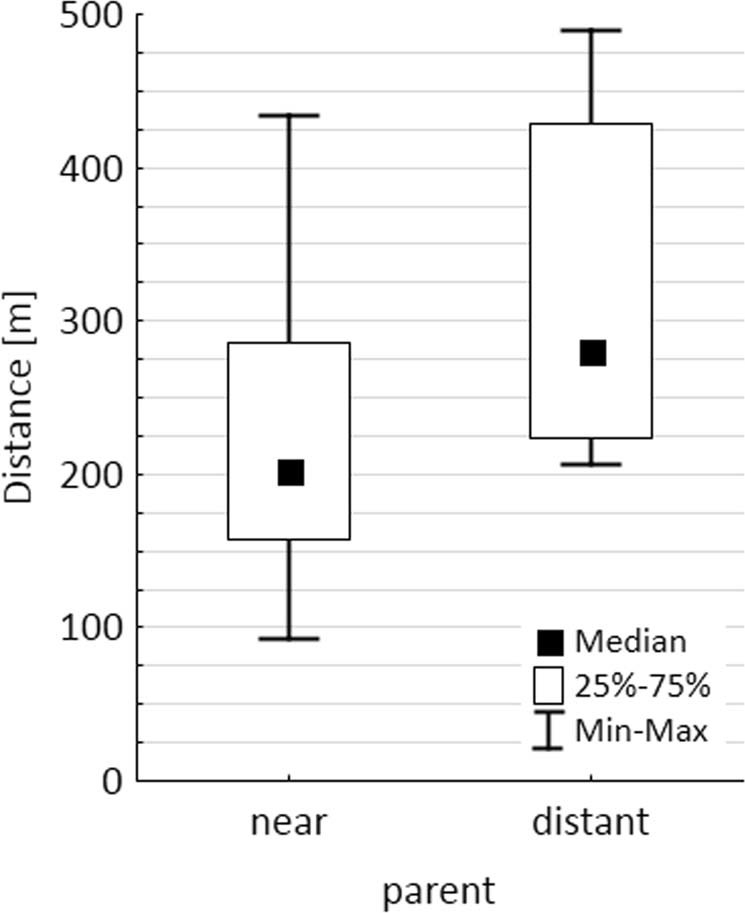
Distances between *Parkia panurensis* seedlings and juveniles and the near and distant candidate parent.

We could match 19 out of 37 (=51%) genotyped *P. panurensis* seedlings and juveniles from the secondary forest to 11 candidate parents in the primary forest (seven seedlings/juveniles with 95% confidence of parent assignment, 12 seedlings/juveniles with 80% confidence) (Fig. 4 and Supplementary Information [Supplementary Table 2]). Distances between seedlings/juveniles and the near candidate parent varied between 92 and 432 m (median: 202 m), distances to the distant candidate parent between 207 and 488 m (median: 281 m; Fig. 5).

## Discussion

The tamarins first entered the disturbed area in July 2000, while it was still sporadically used by water buffaloes. This suggests that the previous non-use of the areas was not due to the presence of the water buffaloes, but that only by 2000 had the vegetation reached a degree of regeneration which allowed the tamarins to use the area. Use of the secondary forest increased over time, but two peaks stick out: 2006 and 2011–12. These are years that followed extreme droughts in Amazonia in 2005 and 2010, respectively^23,24^. Plants growing in relatively open areas and forest edges with drier conditions are more resistant to droughts than plants in the forest interior^25–27^. Therefore, after extreme droughts, their fruit production is more likely to persist in a relatively young secondary forest than in the forest interior. The first observed entry into the secondary forest in a relatively dry month (July) and the general trend for using the secondary forest more often during months with less rainfall is consistent with this reasoning. Tamarins increase their consumption of fruits from pioneer species from the secondary forest like *Cecropia sciadophylla*, *Cecropia distachya* or *Bellucia pentamera* during drier periods of the year with reduced overall fruit availability^19^. While the secondary forest obviously provided fruit resources that attracted the tamarins, up to now they have not been seen sleeping in this area. Apparently, the secondary forest does not yet provide the structures and species used by tamarins for sleeping^28,29^.

Until 2005, most plant species consumed in the secondary forest were small-seeded pioneer species. Then, the tamarins started to consume larger seeded non-pioneer species (e.g., *Mendoncia* sp.: seed length 1.9 cm, width 0.8 cm)^17^. The increasing time spent and diversification of fruit consumption in the secondary forest probably reflect different stages of regeneration, with an increasing habitat quality for tamarins. The longer time spent in secondary forest also increases the probability of seed dispersal seeds into this area, including of seeds coming from the primary forest. Culot and co-workers^19^ showed with data from the same tamarin study group that the quantity of non-pioneer seeds dispersed into the secondary forest increases with the duration of entries and distance travelled in the regenerating habitat. Therefore, since the time spent in secondary forest increases over years, we expect an increasing contribution of tamarins to the regeneration of this forest.

Seeds dispersed by tamarins into the secondary forest resulted in a higher success of seedling establishment compared to seeds dispersed within the primary forest. Differences in plant recruitment might be due to a higher availability of light in secondary forest, lower competition or predation pressure, or a combination of these factors. Specific experiments would be necessary to disentangle these potential effects. The crucial point here is that seeds from the primary forest were dispersed into, and that seedlings recruited and established in, the secondary forest. In addition, the presence of *P. panurensis* juveniles ( > 2 m) confirms the effectiveness of the seed dispersal service provided by tamarins.

Tamarins dispersed *P. panurensis* seeds over distances between 0 and 656 m, and > 90% of seeds land within 350 m of the source tree^30,31^. Distances between the seedlings in the secondary forest and their candidate parents were within the range of dispersal distances, but the medians are located to the right of the peak of dispersal curves (compare with Fig. 5 in^31^. This could be a sample size effect, as dispersal curves with lower sample sizes also show a peak shift towards higher distances (see Fig. 5 in^31,32^). However, it can also result from the fact that tamarins visit the secondary forest preferably in the morning before 0800 h^20^. The travel speed of tamarins is highest in the early morning^33^. Thus, seeds swallowed during feeding bouts in the first hours after leaving the sleeping site have a higher probability of being dispersed over larger distances.

## Conclusion

Our study demonstrates that tamarins may enter an anthropogenically disturbed area in an early stage of regeneration and increase the use of the developing secondary forest over time. They disperse seeds from plants growing in primary forest into the secondary forest. Seeds not only survive for considerable periods of time, but also germinate and produce seedlings and juveniles. Tamarins can thus contribute to the natural regeneration of anthropogenically disturbed areas.

The findings reported here are mainly the outcome of an unplanned study, as in the earlier years of our studies at EBQB we did not anticipate that the disturbed area would regenerate and that the tamarins would ever start to use it. However, our collecting of basic behavioural and ecological data over a long time period, combined with specific research questions and pertinent data collection implemented later allowed us to evaluate the role of seed dispersers on the regeneration process of a disturbed area and the restoration success^34^. Indeed, the study of the behaviour of a key member of the ecosystem (e.g., seed disperser) can provide valuable information about their role in forest regeneration and the critical resources that make a site suitable^34^. In this sense, it is interesting to note that, even after almost 20 years, a secondary forest is not yet sufficient to fulfil all ecological needs of the tamarins which still depend on the primary forest matrix, for example to find suitable sleeping trees.

## Methods

### Study site

**Figure 6:**
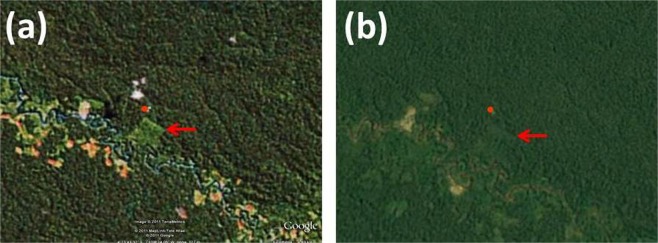
Satellite image of EBQB and surrounding areas. : location of the field station; : secondary forest area. (**a**) 2011; (**b**) 2018. Source: Google Earth.

**Figure 7:**
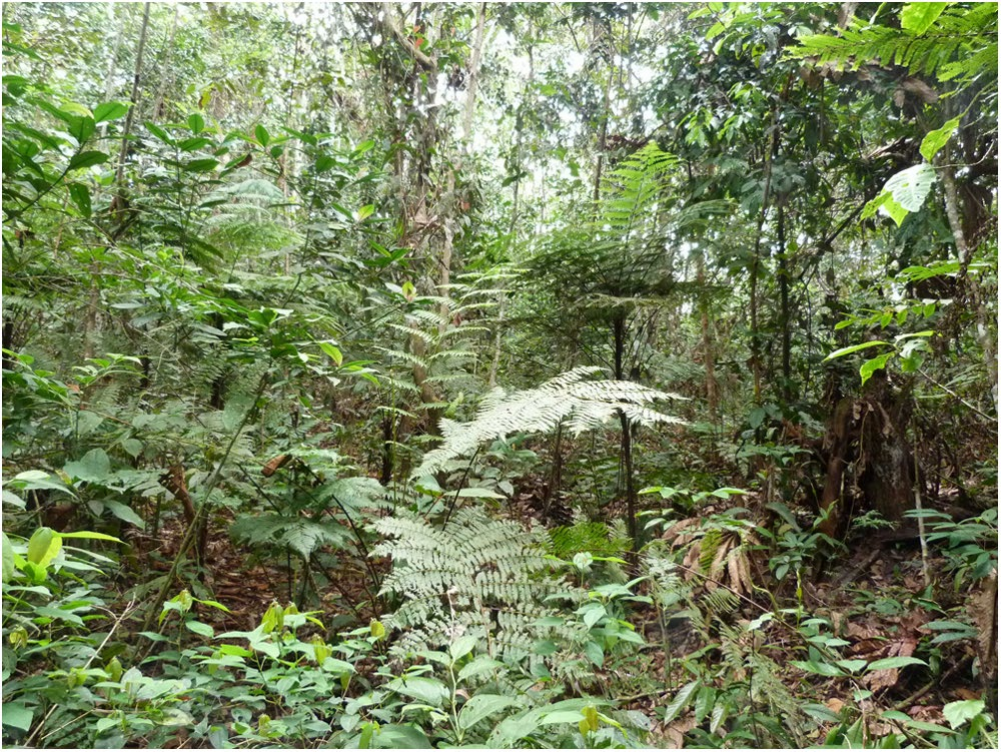
Typical vegetation in secondary forest at EBQB. Foto ©Eckhard W. Heymann, 2010.

**Figure 8:**
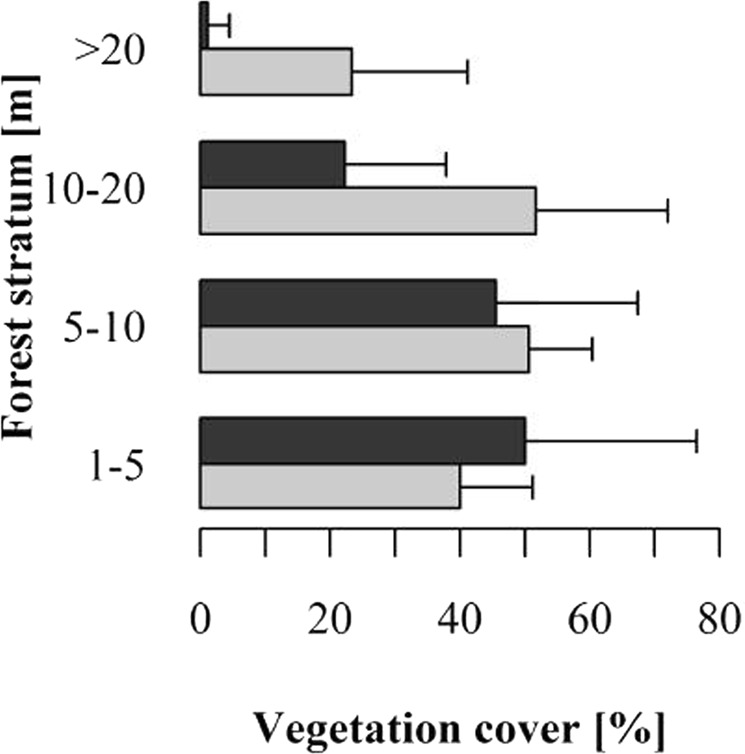
Vegetation cover of primary and secondary forest at EBQB. Grey bars: primary forest; black bars: secondary forest. Reproduced with permission from^43^.

Our studies were conducted at the Estación Biológica Quebrada Blanco (EBQB) in north-eastern Peruvian Amazonia (4°21′S 73°09′W). The major part of the study area is covered by primary rain forest. In the south-eastern part, it includes an area of ca. 4 ha that was completely logged in 1990 and used intermittently as a water buffalo pasture (*Bubalus bubalis*) until 2000 (Fig. 6). Regeneration (without human intervention) already started while the area was still used by buffaloes, particularly at the border to the primary forest, and seemed to speed up after abandonment as buffalo pasture in 2000. The vegetation in the secondary forest is more open than in primary forest (Figs 7, 8), but vegetation cover has increased over the years (Fig. 6). For further details of the study site see^17,28^. Rainfall shows a strongly seasonal pattern, with ≥250 mm/month between December and May, and <250 mm/month between June and November (Supplementary Information [Supplementary Figure]).

### Study species and field methods

Mixed-species groups of *L. nigrifrons* and *S. mystax* are routinely observed at EBQB in the course of behavioural and ecological projects. During all-day follows, the position of groups is recorded with GPS every 10, 15 or 20 minutes, depending on the specific study. In pre-GPS times (from 1995 to 2003), positions were recorded by using the 100 m x 100 m trail grid system (covering ca. 1 km²) and mapped plants as reference points. Only one of the routinely observed groups regularly enters the secondary forest. This group was observed for variable periods of time between 1994 and 2016 (see Supplementary Dataset). The home-range areas of two other routinely observed groups did not border the secondary forest. Another group was seen in the central and southern/south-eastern part of the secondary forest during intergroup encounters with the study group of this report.

Feeding behaviour of the mixed-species group was studied in 1994–1995, 2000 and 2005–2008. All feeding bouts (defined as the period when one or several group members were feeding in a fruiting plant) were recorded for both species using the all-occurrence method^35^. We registered the tamarin species present, the time of entry into the fruiting plant, the spatial location, the life form (tree, vine, epiphyte/hemi-epiphyte), and the plant species.

We registered the spatial location of seeds defecated by tamarins once a week, three to four weeks per month between 2005 and 2008, identified them and followed their fate for at least one year (more details about the method can be found in^36^).

*Parkia panurensis* (Fabaceae: Mimosoideae) is widely distributed in western and central Amazonia, mainly found in terra firme-forests and reaching up to 35 m height^37^. At EBQB, adult *P. panurensis* are found in primary forest at a density of ca. 1 tree/ha. It is a major food plant of tamarins who routinely and consistently disperse the seeds of this plant over distances up to 700 m, but mainly within 300 m^15,38^. Diurnal and nocturnal focal tree observations provided evidence that tamarins are the exclusive seed dispersers of *P. panurensis* at EBQB. Additional information on *Parkia* is provided in the Supplementary Information (Supplementary Material 1).

To examine whether seedlings and juveniles of *P. panurensis* in the secondary forest are the product of tamarin seed dispersal, we collected leaves from 37 seedlings and juveniles in the secondary forest (Fig. 3), and dried and stored them on Silica Orange for subsequent genotyping. We also recorded the height and GPS position of these seedlings and juveniles.

### Statistical methods

To analyse the long-term patterns in use of the regenerating area, we used monthly values for the percentage of the activity period spent in the secondary forest. From these data, we modelled the yearly trend. First, we imputed missing values by interpolating the data with a Kalman filter, followed by a smoothing procedure^39^. Estimation of the Kalman filter was done via maximum likelihood estimation, using the function StructTS from the package xts. Visual inspection of the data suggested the existence of monthly trends and a seasonal component. Therefore, in a second step, we applied a classical additive decomposition of the time series into a trend, a seasonal component and an error term, using moving average smoothing^40,41^. The decomposition was obtained from the following procedure: the trend was first estimated using moving average smoothing and removed, before estimating the seasonal component as the mean over all observation for a given time unit, using the function decompose of the package xts. Finally, we computed the yearly trend by averaging all monthly trend estimates from the same year. All trend analyses were performed with R 3.4.3; the codes are provided in the Supplementary Information (Supplementary Material 2).

To examine monthly variation, we performed a 1-way ANOVA with month as categorical monthly variable and percentage of the activity period spent in the secondary forest as response variable. We also compared rainy season months (≥250 mm rainfall; December-May) and “dry” season months (<250 mm rainfall (June-November) with a t-test. Since the secondary forest was first used by the tamarins in 2000, we excluded data from previous years from these analyses. Both the ANOVA and the t-test were performed in Statistica 13^42^.

### Genetic methods and parentage analyses

Genotyping of seedlings and juveniles followed the protocol described in Heymann *et al*. (2012). Genotypes of candidate parent trees from the primary forest (no adult *P. panurensis* trees are found in the secondary forest) were available from two studies by Heymann *et al*.^30^ and^38^. Of the 37 seedlings, 34 could be genotyped successfully and used in the parentage analysis with CERVUS 3.0.7 (http://www.fieldgenetics.com/pages/aboutCervus_Overview.jsp). Parent sex was set to unknown (as any *P. panurensis* can be both mother and father) and the proportion of known candidates to 70%. Distances between seedlings and the two candidate parents (confidence level for parentage assignment >80%) were calculated online at https://rechneronline.de/geo-koordinaten. We then calculated the median distances between seedlings and the near and distant candidate parent, respectively.

### Meteorological data

We compiled data on monthly rainfall from the nearest meteorological station (Tamshiyacu, 4°00′10.7″S, 73°09′38.2″W, 40 km north of EBQB) provided by the Servicio Nacional de Meteorología e Hidrología del Perú (http://www.senamhi.gob.pe/main_mapa.php?t=dHi).

## Supplementary information

Supplementary Information

Supplementary Dataset
