# Author Correction: Small Neotropical primates promote the natural regeneration of anthropogenically disturbed areas

**DOI:** 10.1038/s41598-020-57958-z

**Published:** 2020-01-27

**Authors:** Eckhard W. Heymann, Laurence Culot, Christoph Knogge, Andrew C. Smith, Emérita R. Tirado Herrera, Britta Müller, Mojca Stojan-Dolar, Yvan Lledo Ferrer, Petra Kubisch, Denis Kupsch, Darja Slana, Mareike Lena Koopmann, Birgit Ziegenhagen, Ronald Bialozyt, Christina Mengel, Julien Hambuckers, Katrin Heer

**Affiliations:** 1 Verhaltensökologie & Soziobiologie, Deutsches Primatenzentrum – Leibniz-Institut für Primatenforschung, Göttingen, Germany; 2 Laboratório de Primatologia, Departamento de Zoologia, Universidade estadual Paulista - UNESP, Rio Claro, SP Brazil; 3 Primatology Research Group, Behavioral Biology Unit, University of Liège, Liège, Belgium; 4 School of Life Sciences, Anglia Ruskin University, Cambridge, UK; 5 Facultad de Ciencias Biológicas, Universidad Nacional de la Amazonía Peruana, Iquitos, Peru; 6 Present Address: Bayerisches Landesamt für Gesundheit und Lebensmittelsicherheit, Erlangen, Germany; 7 Facultad de Psicología, Universidad Autónoma, Madrid, Spain; 8 Present Address: Albrecht-von-Haller-Institut für Pflanzenwissenschaften, Abteilung Ökologie & Ökosystemforschung, Georg-August Universität Göttingen, Göttingen, Germany; 9 Present Address: Naturschutzbiologie, Georg-August Universität Göttingen, Göttingen, Germany; 10 Present Address: Bioplan Marburg, Marburg, Germany; 11 Naturschutzbiologie, Philipps-Universität, Marburg, Marburg, Germany; 12 Present Address: Nordwestdeutsche Forstliche Versuchsanstalt, Göttingen, Germany; 13 Chair for Statistics and Econometrics, Georg-August Universität Göttingen, Göttingen, Germany; 14 Present Address: Department of Finance, HEC Liège, University of Liège, Liège, Belgium

Correction to: *Scientific Reports* 10.1038/s41598-019-46683-x, published online 25 July 2019

In the original HTML version of this Article, Christina Mengel and Katrin Heer were incorrectly affiliated with ‘Nordwestdeutsche Forstliche Versuchsanstalt, Göttingen, Germany’. Their correct affiliation is listed below:

‘Naturschutzbiologie, Philipps-Universität, Marburg, Germany’

This error has now been corrected in the HTML version of the Article, and in the accompanying Supplementary Information file; the PDF was correct at the time of publication.

