# Supplementary material for: Small Neotropical primates promote the natural regeneration of anthropogenically disturbed areas
Source: Sci Rep. 2019 Jul 25;9:10356. doi: 10.1038/s41598-019-46683-x (PMC6658533; doi:10.1038/s41598-019-46683-x)
Supplement: Supplementary file 1 — Supplementary Information [file 41598_2019_46683_MOESM1_ESM.pdf]

Small Neotropical primates promote the natural regeneration of anthropogenically disturbed areas

Eckhard W. Heymann[1,*], Laurence Culot[1,2,3,*], Christoph Knogge[1], Andrew C. Smith[4], Emérita R. Tirado Herrera[1,5], Britta Müller[1,6], Mojca Stojan-Dolar[1], Yvan Lledo Ferrer[1,7], Petra Kubisch[1,8], Denis Kupsch[1,9], Darja Slana[1], Mareike Lena Koopmann[1,10], Birgit Ziegenhagen[11], Ronald Bialozyt[11,12], Christina Mengel[12], Julien Hambuckers[13,14] & Katrin Heer[12]

[1] Verhaltensökologie & Soziobiologie, Deutsches Primatenzentrum – Leibniz-Institut für Primatenforschung, Göttingen, Germany

[2] Laboratório de Primatologia, Departamento de Zoologia, Universidade Estadual Paulista - UNESP, Rio Claro, SP, Brazil

[3] Primatology Research Group, Behavioral Biology Unit, University of Liège, Liège, Belgium

[4] School of Life Sciences, Anglia Ruskin University, Cambridge, UK

[5] Facultad de Ciencias Biológicas, Universidad Nacional de la Amazonía Peruana, Iquitos, Peru

[6] current affiliation: Bayerisches Landesamt für Gesundheit und Lebensmittelsicherheit, Erlangen, Germany

[7] Facultad de Psicología, Universidad Autónoma Madrid, Spain

[8] current affiliation: Albrecht-von-Haller-Institut für Pflanzenwissenschaften, Abteilung Ökologie & Ökosystemforschung, Georg-August Universität Göttingen, Germany

[9] current affiliation: Naturschutzbiologie, Georg-August Universität Göttingen, Germany

[10] current affiliation: Bioplan Marburg, Marburg, Germany

[11] Naturschutzbiologie, Philipps-Universität Marburg, Germany

[12] current affiliation: Nordwestdeutsche Forstliche Versuchsanstalt, Göttingen, Germany

[13] Chair for Statistics and Econometrics, Georg-August Universität Göttingen, Germany

[14] current affiliation: Department of Finance, HEC Liège, University of Liège, Belgium

Supplementary Figure.

Monthly variation of rainfall at Tamshiyacu (4°00’10.7”S 73°09’38.2”W), ca. 40 km north of EBQB, between 1997 and 2016.

Means (●) ± 95% confidence intervals (whiskers). Raw data downloaded from http://www.senamhi.gob.pe

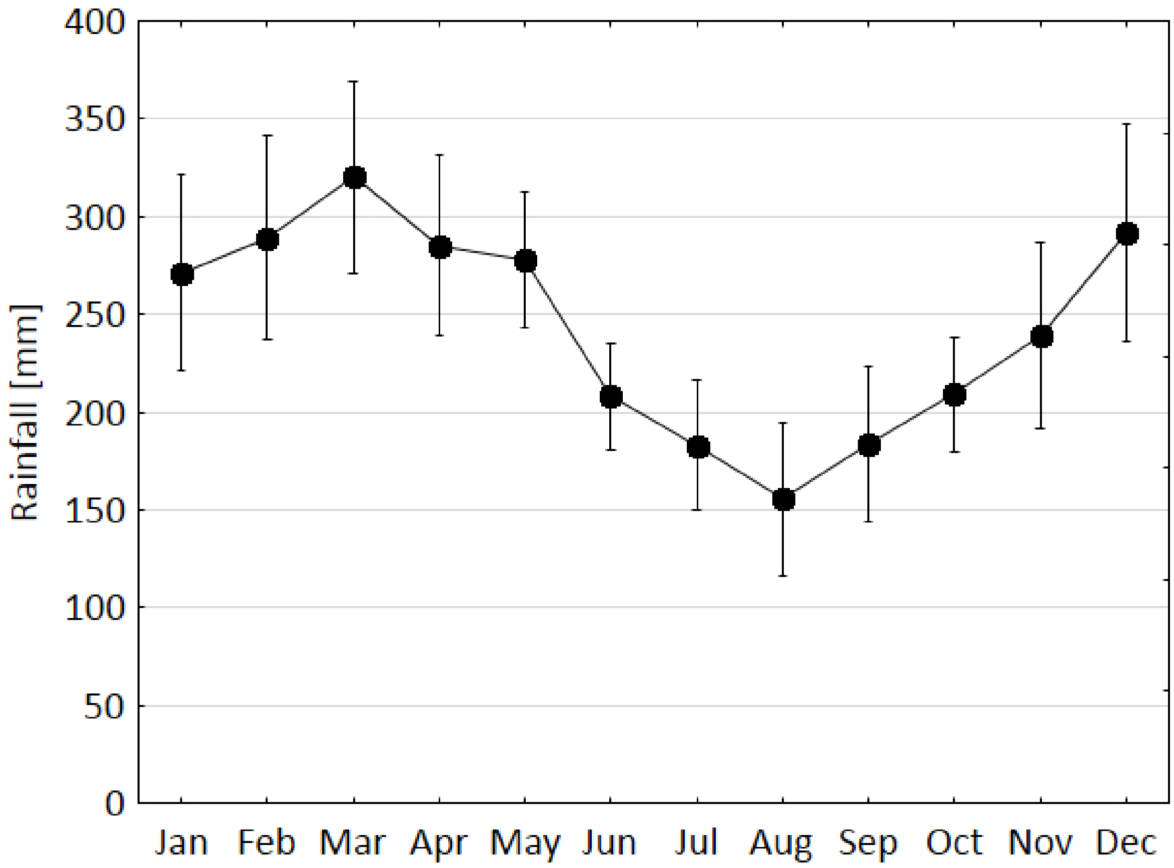

Supplementary Table 1.

Plant species exploited for food by the tamarins in secondary forest.

Species in bold letters are dispersed by the tamarins.

Family	Species	Life form
Acanthaceae	***Mendoncia klugii***	liana
Acanthaceae	***Mendoncia* sp.**	liana
Anacardiaceae	***Tapirira guianensis***	tree
Annonaceae	***Annona duckei***	tree
Annonaceae	*Xylopia* sp.*	tree
Apocynaceae	***Lacmellea peruviana***	tree
Cecropiaceae	***Cecropia distachya***	tree
Cecropiaceae	***Cecropia sciadophylla***	tree
Fabaceae	***Inga auristellae***	tree
Fabaceae	***Inga brachyrhachis***	tree
Fabaceae	***Inga lopadadenia***	tree
Fabaceae	***Inga megaphylla***	tree
Fabaceae	***Inga oerstediana***	tree
Fabaceae	***Inga* sp. 4**	tree
Flacourtiaceae	***Casearia pitumba***	tree
Loganiaceae	***Strychnos rondeletioides***	liana
Malpighiaceae	***Byrsonima poeppigiana***	tree
Malpighiaceae	*Byrsonima stipulina*†	tree
Melastomataceae	*Miconia ternatifolia*‡	tree
Melastomataceae	***Tococa guianensis***	tree
Menispermaceae	***Telitoxicum minutiflorum***	liana
Moraceae	***Ficus americana***	tree

Moraceae	***Ficus krukovii***	tree
Polygalaceae	***Moutabea aculeata***	liana
Rubiaceae	***Cordia nodosa***	tree
Rubiaceae	***Cordia trachyphylla***	tree
Rubiaceae	***Pentagonia spathicalyx***	tree
Vitaceae	***Cissus biformifolia***	liana
indetermined	NI 8	liana
indetermined	NI 9	liana

[*] Seeds of *Xylopia* are generally dispersed endozoochorously [1].

[†] No information on seed dispersers for this species available. Many other species of the genus are dispersed endozoochorously by birds [1].

[‡] Endozoochorously dispersed by birds [2].

Reference

1 van Roosmalen, M. G. M. *Fruits of the Guianan Flora*. (Institute of Systematic Botany, 1985).

2 Gorchov, D. L., Cornejo, F., Ascorra, C. F. & Jaramillo, M. Dietary overlap between frugivorous birds and bats in the Peruvian Amazon. *Oikos* **74**, 235-250 (1995).

Supplementary Table 2.

Seedlings, candidate parents, UTM data (UTM zone 18) and trio confidence for parentage assignment. *: 95% confidence, +: 80% confidence

Seedling	Location of seedling		First candidate parent	Location of first candidate parent		Second candidate parent	Location of second candidate parent		Trio confidence
	easting	northing		easting	northing		easting	northing	
SF 13	704296,38	9517273,42	M 13	704035,85	9517686,18	M 20	704088,17	9517651,77	*
SF 14	704296,38	9517273,42	M 13	704035,85	9517686,18	M 20	704088,17	9517651,77	+
SF 01	704385,38	9517350,62	M 13	704035,85	9517686,18	M 50	704032,73	9517590,86	+
SF 02	704363,09	9517313,81	M 15	704070,42	9517247,70	M 42	704129,60	9517208,52	+
SF 08	704290,85	9517282,65	M 15	704070,42	9517247,70	M 42	704129,60	9517208,52	+
SF 17	704300,05	9517258,67	M 15	704070,42	9517247,70	M 42	704129,60	9517208,52	+
SF 29	704307,52	9517289,99	M 15	704070,42	9517247,70	M 42	704129,60	9517208,52	*
SF 30	704307,52	9517289,99	M 15	704070,42	9517247,70	M 42	704129,60	9517208,52	+
SF 16	704296,38	9517271,58	M 15	704070,42	9517247,70	M 61	704165,27	9517222,26	+
SF 31	704274,21	9517284,54	M 15	704070,42	9517247,70	M 61	704165,27	9517222,26	+
SF 33	704274,21	9517284,54	M 15	704070,42	9517247,70	M 61	704165,27	9517222,26	*
SF 34	704274,33	9517334,30	M 15	704070,42	9517247,70	M 61	704165,27	9517222,26	+
SF 21	704296,51	9517326,88	M 18	704372,34	9517734,02	M 41	704421,68	9517571,88	*
SF 22	704289,08	9517315,84	M 18	704372,34	9517734,02	M 46	704004,83	9517347,95	*
SF 26	704329,93	9517372,88	M 37	704324,50	9517464,73	M 61	704165,27	9517222,26	*
SF 05	704311,24	9517295,51	M 42	704129,60	9517208,52	M 46	704004,83	9517347,95	+
SF 20	704285,45	9517341,65	M 42	704129,60	9517208,52	M 46	704004,83	9517347,95	+
SF 07	704294,59	9517297,39	M 50	704032,73	9517590,86	M 55	703980,49	9517518,32	+
SF 19	704301,83	9517232,86	M 50	704032,73	9517590,86	M 55	703980,49	9517518,32	*

Supplementary Material 1.

Information on Neotropical *Parkia*

The genus *Parkia* (family Fabaceae, subfamily Mimosoideae) has a pan-tropical distribution [1,2]. In the Neotropics, it is represented by 19 or more species that mostly grow as medium to tall or even emergent trees in terra firme forests [1,3,4]. The compound inflorescences are pollinated by bats and bees [1,5]. Seeds of Neotropical *Parkia* are mainly dispersed by primates (*Ateles belzebuth*, *Lagothrix cana*, *Lagothrix poeppigii*, *L. nigrifrons*, *S. mystax*) and terrestrial rodents [6-10]. Known seed predators include bruchid beetles, ants, large parrots, primates (*Cacajao calvus ucayalii, Sapajus macrocephalus, Pithecia albicans*), and terrestrial ungulates and rodents [6,8,11,12] [own observations].

References

1 Hopkins, H. C. *Parkia* (Leguminosae, Mimosoideae). *Flora Neotropica* **43**, 1-124 (1986).

2 Luckow, M. & Hopkins, H. C. F. A cladistic analysis of *Parkia* (Leguminosae: Mimosoideae). *American Journal of Botany* **82**, 1300-1320 (1995).

3 Neill, D. A. *Parkia nana* (Leguminosae, Mimosoideae), a new species from the sub-Andean sandstone cordilleras of Peru. *Novon* **19**, 204-208 (2009).

4 Vásquez Martínez, R. *Flórula de las reservas biológicas de Iquitos, Perú*. (Missouri Botanical Gardens, 1997).

5 Hopkins, H. C. Floral biology and pollination ecology of the neotropical species of *Parkia*. *Journal of Ecology* **72**, 1-23 (1984).

6 Peres, C. A. Identifying keystone plant resources in tropical forests: the case of gums from *Parkia* pods. *Journal of Tropical Ecology* **16**, 287-317 (2000).

7 Knogge, C. & Heymann, E. W. Seed dispersal by sympatric tamarins, *Saguinus mystax* and *Saguinus fuscicollis*: diversity and characteristics of plant species. *Folia Primatologica* **74**, 33-47 (2003).

8 Hopkins, H. C. & Hopkins, M. J. G. in *Tropical rain forest: ecology and management* (eds S L Sutton, T C Whitmore, & A C Chadwick) 197-209 (Blackwell Scientific Press, 1983).

9 Culot, L., Huynen, M.-C., Gérard, P. & Heymann, E. W. Short-term post-dispersal fate of seeds defecated by two small primate species (*Saguinus mystax* and *Saguinus fuscicollis*) in the Amazonian forest of Peru. *Journal of Tropical Ecology* **25**, 229-238 (2009).

10 Dew, J. L. in *Spider monkeys. Behavior, ecology and evolution of the genus Ateles* (ed C J Campbell) 155-182 (Cambridge University Press, 2008).

11 Bowler, M. & Bodmer, R. E. Diet and food choice in Peruvian red uakaris (*Cacajao calvus ucayalii*): selective or opportunistic seed predation? *International Journal of Primatology* **32**, 1109-1122 (2011).

12 Feldmann, M., Verhaagh, M. & Heymann, E. W. *Sericomyrmex* ants as seed predators. *Ecotropica* **6**, 207-209 (2000).

Supplementary Material 2

R code used in the analyses of the long term trend in the use of the secondary forest

```
rm(list=ls(all=TRUE))

setwd("C:/wd")
library(ggplot2)
library(ggfortify)
library(zoo)
library(xts)
library(gam)


# load the data
data<-read.csv("secondary_forest_final.csv", sep=",", dec=".", header = TRUE)
v<-zooreg(data$percTime, start=as.yearmon("1994-11"), freq=12)
dnew<-na.StructTS(v)
res<-decompose(dnew) #there is a warning but it should not be a problem
res_seasonal<-res$seasonal
res_trend<- res$trend

##  Original time series without Kalman filter  ##
v <- fortify(v) # fortify() creats an data.frame, which is suitable for ggplot
p<-ggplot(data = v, aes(x = Index, y = v)) + geom_line() +
  scale_x_yearmon(format = "%Y")
p <- p + labs(x = "Time")+ labs(y = "% of occupation")+labs(title = "Original time series")

##  with Kalman filter  ##

dnew <- fortify(dnew)
p<-ggplot(data = dnew, aes(x = Index, y = dnew)) + geom_line() +
  scale_x_yearmon(format = "%Y")
p <- p + labs(x = "Time")+ labs(y = "% of occupation")+labs(title = "Reconstructed time series")

## Seasonal component ##

res_seasonal1 <- fortify(res_seasonal)
res_seasonal1$Month <- format(as.Date(res_seasonal1$Index), "%m")
res_seasonal1<-res_seasonal1[3:14,]
res_seasonal1$Month2<-
c("Jan","Feb","Mar","Apr","Mai","June","July","Aug","Sep","Oct","Nov","Dec")
p<-ggplot(data = res_seasonal1, aes(x = Month, y = Data, group = 1)) +  geom_path()+ labs(x =
"Month")+ labs(y = "% of occupation")+labs(title = "Seasonal component")
p <- p + labs(x = "Month")+ labs(y = "% of occupation")+labs(title = "Seasonal component")

## Trend component ##
```

```
res_trend <- fortify(res_trend)
p<-ggplot(data = res_trend, aes(x = Index, y = Data)) + geom_line()
p <- p + labs(x = "Time")+ labs(y = "% of occupation")+labs(title = "Trend component")

### Yearly average of the trend

y <- rep(NA, times = 22)

for (i in 1:22){
  y[i]<-mean(na.omit(res_trend$Data[(3+12*(i-1)):((14)+12*(i-1))]))
}

df <- data.frame(x = seq(from = 1995, to = 2016), y = y)
p <- ggplot(data = df, aes(x = x, y = y)) + geom_line() +
  labs(x = "Time", y = "% of occupation", title = "Average trend")
plot(p)
```
